# Recent advances in epilepsy

**DOI:** 10.1007/s00415-017-8394-2

**Published:** 2017-01-24

**Authors:** Mark Manford

**Affiliations:** Department of Clinical Neurosciences, Addenbrooke’s Hospital and University of Cambridge, Hills Rd, Cambridge, CB2 0QQ UK

**Keywords:** Epilepsy, Classification, Status epilepticus, Treatment, Pregnancy, Epileptogenesis

## Abstract

This paper reviews advances in epilepsy in recent years with an emphasis on therapeutics and underlying mechanisms, including status epilepticus, drug and surgical treatments. Lessons from rarer epilepsies regarding the relationship between epilepsy type, mechanisms and choice of antiepileptic drugs (AED) are explored and data regarding AED use in pregnancy are reviewed. Concepts evolving towards a move from treating seizures to treating epilepsy are discussed, both in terms of the mechanisms of epileptogenesis, and in terms of epilepsy’s broader comorbidity, especially depression.

## Definitions and classification

Definitions in epilepsy have always been problematic [1–5]. The disorder is characterised by seizures but not all seizures are due to epilepsy—febrile seizures or drug induced seizures, for example. Earlier classifications sought to reconcile these difficulties by describing different electroclinical syndromes but new data from modern imaging and genetics need to be incorporated.

Diagnosis is difficult because in practice, the diagnostic electrical hallmark of epilepsy may be absent interictally, especially in adults or if seizures are infrequent and interictal epileptiform discharges may occasionally be present in those without seizures. Moreover, in some instances, an “epileptic EEG” may be associated with an epileptic encephalopathy, in which overt seizures may be few or none, such as Landau–Kleffner syndrome, and a cognitive disorder dominates the presentation.

The International League Against Epilepsy recently consulted in an attempt to synthesise a consensus view [6], whose output will be published in 2017. The result promises to be useful and pragmatic, recognizing that the syndromes are multifaceted; any one case defined by an association of clinical, electrophysiological, etiological and comorbid factors. It also accepts that it is not always known if seizures are part of focal or generalized epilepsy and that in some cases, such as tuberous sclerosis, genetic and structural causes overlap. Some terms will be dropped, for example, childhood epilepsies where the seizures remit will be called pharmacoresponsive rather than benign, recognizing that children whose seizures remit may nevertheless have significant persisting psychosocial comorbidities.

The ILAE has also pondered the question of whether a single seizure may be considered to be epilepsy [7] and concluded that it may if there is a greater than 60% chance of another seizure; a risk conferred by the presence of EEG spikes or a major structural aetiology. Epilepsy may be considered to have gone away after ten years with no seizures and with no treatment. This approach has pragmatic utility, rather than mechanistic validity and is useful in allowing driving regulatory authorities to treat those with lower risk more leniently and may be helpful in deciding when to treat medically after a single seizure [8].

Some frontal lobe epilepsies may be particularly difficult to diagnose, often with non-diagnostic ictal scalp EEGs and some were initially considered to be a movement disorder, e.g. “paroxysmal nocturnal dystonia” [9] in which its epileptic basis was shown later [9, 10]. The situation has become more complex with the discovery that patients with frontal lobe epilepsy may also have epileptic nocturnal wandering, with similarities to parasomnias and also brief nocturnal movements which are not due to seizure discharges but may be a release phenomenon of interictal discharges [11]. They may suffer also from non-epileptic parasomnias more frequently than the general population. In the new classification, the phenomenon will be renamed “Sleep-related hypermotor epilepsy (SHE)”.

## Status epilepticus and limbic encephalitis

The ILAE recently defined status epilepticus as: “a condition resulting either from the failure of the mechanisms responsible for seizure termination or from the initiation of mechanisms, which lead to abnormally, prolonged seizures (after time point *t*_1_). It is a condition, which can have long-term consequences (after time point *t*_2_), including neuronal death, neuronal injury, and alteration of neuronal networks, depending on the type and duration of seizures” [12]. Timepoint *t*_1_ is at 5 min after seizure onset, when it is recognized for generalized tonic–clonic status epilepticus that evolution to status is increasingly likely and when treatment should be initiated. T2 is at 30 min, after which there is increasing risk of irreversible consequences. Status is divided along four axes; semiology, aetiology, EEG correlates and age. These axes align with the prognosis of status, which when adequately treated is determined by cause and the age and gender of the patient. The electroclinical state is another prognosticator; subtle status evolving from convulsive status has a particularly poor prognosis [13, 14].

The impressive out-of-hospital randomized, double-blind RAMPART study has shown that IM midazolam is at least as effective as IV lorazepam in the early treatment of status, in adults and children [15, 16], probably because IM speed of administration of midazolam compensates for speed of IV distribution of lorazepam. It has long been known that the effect of benzodiazepines in status epilepticus wears off very rapidly [17, 18] and it has subsequently been demonstrated that GABA_A_ receptor sensitivity is reduced, sometimes long term [18]. Receptor trafficking may be contributory [19, 20]. As well as a reduction in inhibitory neurotransmitters, within 1 h of onset of status in rats, there is an increase in surface NMDA receptors in status, associated with increased excitation [21]. Cholinergic mechanisms are also implicated, supported by the observation that in pilocarpine induced status epilepticus; the addition of scopolamine provides additional seizure control, when combined with phenobarbital and benzodiazepines, raising the possibility of the use of drug combinations in status [22].

Basic mechanisms are starting to align with clinical evidence in the initial treatment of status with benzodiazepines, but thereafter the evidence is less clear. Initial uncontrolled reports suggested a 70% success rate for the treatment of status epilepticus with levetiracetam [23], but a recent randomized controlled trial of out-of-hospital clonazepam plus either levetiracetam or placebo was abandoned because of a lack of benefit in the levetiracetam arm [24]. This mirrors the finding that diazepam plus phenytoin confers no additional benefit to lorazepam alone at 12 h [14] and raises questions around the appropriate timing of the addition of AED to benzodiazepines. It also emphasizes the importance of properly controlled studies in an area where few have been undertaken. Shorvon et al. have undertaken meta-analyses of existing therapies [25–27]. From generally poor quality studies of lacosamide, levetiracetam, phenobarbital, phenytoin or valproate in benzodiazepine resistant status, they found efficacy ranging from 50% (phenytoin), to levetiracetam (68.5%), phenobarbital (58–84%) and valproate 76%. Lacosamide treatments were too few to give figures. The conclusion remains that all these drugs may be useful but there is no clear guidance on choice. The caution with which data from uncontrolled studies must be interpreted is highlighted by a recent randomized study of valproate versus phenobarbital which showed a 44% response to valproate and an 81% response to phenobarbital. However, in children, valproate may have fewer adverse effects and better efficacy than phenobarbital [28, 29] and similar efficacy to phenytoin [30]. But children may not be comparable to adults with a greater proportion of generalized epilepsies, more responsive to valproate. Future options include derivatives of valproate such as valnactomide and butylpropylacetamide, which may be more potently antiepileptic and less teratogenic in animal studies [31].

For status epilepticus which remains refractory to a second line AED, a range of intravenous benzodiazepines or anaesthetic agents may be considered and again Shorvon et al. found that studies are of poor quality. They found that 35% of patients in these studies died and a further 13% had severe neurological deficits and 13% mild neurological deficits on recovery. Studies underway may help answer some of these questions [32, 33]. Ketamine’s role in blocking NMDA receptors [34] has led to it become increasingly popular in the treatment of refractory status, with some efficacy on the basis of uncontrolled retrospective series [35–37]. A randomized trial in children is planned [38]. A recent trial of hypothermia showed no benefit at 90 days [39].

It is increasingly recognized that some patients with refractory status epilepticus, where the cause was previously unrecognized, may be suffering from an antibody mediated encephalopathy, “limbic encephalitis”. Antibodies implicated include LGI1 and NMDA, with CASPR less associated with seizures [40, 41]. More recently, GABA_B_ and AMPA receptors have been implicated in some cases [42]. A specific phenotype of very brief, frequent and highly focal, faciobrachial dystonic seizures is almost pathognomonic of LGI1 associated disease, often heralding a more severe encephalopathy [43] and providing an opportunity to intervene at an earlier stage. Limbic encephalitis exhibits characteristic changes on MRI in the mesial temporal structures, especially the amygdalae [44] and responds primarily to immunotherapy and treatment of any associated tumour, rather than to AED [41, 45]. Early suspicion of the diagnosis and treatment, even before definitive serological confirmation, is recommended. Many patients will recover with appropriate treatment but may be left with ongoing epilepsy and hippocampal sclerosis is a reported outcome [46]. The extent to which epilepsy in patients, who have not suffered limbic encephalitis, may be attributable to antibody-mediated disease is an area of exploration which may open new avenues of treatment for chronic epilepsy. Small cohorts suggest increased rates of antibody positivity but their significance is not yet clear [47, 48].

## Pharmacological treatment of epilepsy and underlying mechanisms/genetics

In 2000, Kwan and Brodie [49] found that 63% of unselected patients in an epilepsy service were rendered seizure free with medication. Since then despite numerous antiepileptic drugs becoming available, they found that the chance of a patient, who is diagnosed in 2017 becoming seizure free, has changed little [50]. Some studies are more optimistic; refractory epilepsy may have a greater chance of 12-month remission with or without AED change [51–53] at around 5% per year and although up to 40% may relapse [51], many of these may have a second longer remission.

The broad sweep of AEDs, generally affecting ion channels or neurotransmitters is unchanged, but there is slowly increasing evidence for a differential effect in specific syndromes.

Of established epilepsy drugs, ethosuximide, often forgotten by adult neurologists, has the most specific mechanism in relation to its role in the absence epilepsy. It acts on T-type calcium channels [54], implicated in the thalamocortical disturbance believed for decades to underlie generalized epilepsies [55]. Valproate and ethosuximide have clearly demonstrated greater efficacy over lamotrigine in childhood absence epilepsy [56]. A small, non-randomized study has suggested that ethosuximide may be also associated with a greater chance of long-term remission [57]. In a mouse model of absence epilepsy, Bomben et al. [58] selectively ablated P/Q channels in the neurons of layer VI that provide the descending cortical projection to the thalamus. This produced spike-wave activity with clinical absences suppressed by ethosuximide. This very selective lesion supports the view that a highly specific cortical abnormality is necessary and sufficient to generate the thalamocortical oscillations of absence epilepsy. Not all patients respond equally to medication. A clinical imaging and EEG study, comparing those patients responsive to valproate to those who are resistant, suggested different patterns of activation may underlie the varying therapeutic responses [59].

Despite strong epidemiological evidence of a genetic basis of IGE, relevant genes remain elusive, hampering efforts to identify specific drug targets. A recent genome wide association study suggested links to SCN1A, a known cause of GEFS+, protocadherin PCDH7 and PCDH19, both known to be associated with epilepsy and learning disability [60]. An analysis of microdeletions in generalized epilepsy showed an increased burden (7.3%) compared to controls (4%) and specific involvement of a range of genes known to be important in epilepsy, psychiatry and neurodevelopment [61].

The first major application of pharmacogenetics in epilepsy, and probably still the most widely applicable, has been the identification of patients from South East Asia who are HLA-B*1502 positive, putting them at high risk for Stevens–Johnson syndrome from carbamazepine and the elimination of this life-threatening complication by pre-treatment screening [62, 63]. Genetic understanding is creeping into other areas of pharmacological therapeutics. It has been realized for a number of years that sodium channel blocking drugs may be deleterious for children with Dravet syndrome [64, 65], although this may not be so clear for adult patients [66]. It is now known that Dravet syndrome is commonly due to a genetic truncations leading to total loss of function or missense mutations causing partial loss of function of the sodium channel, usually SCN1A [67, 68], which is located on inhibitory interneurons and causes hyperexcitability and seizures as a result of loss of function. A previously empirical observation of relative AED efficacy is now underpinned by a mechanistic understanding, which can guide drug choice. Mutations of the SCN8A gene are also associated with epilepsy, sometimes with a Dravet-like syndrome [69]. However, the phenotype may depend on the pathophysiology of the mutation, which may be a gain or a loss of function [70]. In four children with epileptic encephalopathy onset in the first months of life, Boerma described a response to phenytoin [71]. One of these had been demonstrated to have a gain of function mutation.

There are a number of other instances where rare monogenic cases of epilepsy have been evaluated in detail and treatment tailored to the identified pathophysiological mechanism, with varying success. Most consistently effective is the use of ketogenic diet to switch cerebral energy metabolism away from glucose in patients with Glut-1 deficiency, which may be dramatically successful [72, 73]. Retigabine (ezogabine) increases activity at KCNQ2 channels [74] and has been used to treat the neonatal epileptic encephalopathy associated with reduced function mutations of the KCNQ2 channel with some success [75]. Unfortunately, this drug is to be withdrawn from use in 2017 because of the pigmentary changes it may induce in skin, mucosae and eyes [76]. GRIND2 mutations resulting in gain of activity of the NMDA receptor may cause balloon swelling and cell death. Children with a severe encephalopathy due to this mutation may possibly benefit from treatment with memantine, more generally used in Alzheimer’s disease which inhibits this channel [77]. KCNT1 encodes a sodium-activated potassium channel and has been implicated in the migrating partial epilepsy of childhood and in autosomal dominant frontal lobe epilepsy, both causing a gain of function [78]. Two children with this mutation and a severe epilepsy phenotype were helped by the administration of quinidine [79]. These cases illustrate the importance of not only an electroclinical and genetic diagnosis of these epilepsies but also delineation of the specific pathophysiology of the mutation to enable drug choice, which may include opportunities beyond those conventionally used in the antiepileptic armamentarium.

## Epileptogenesis and inflammation

**Fig. 1:**
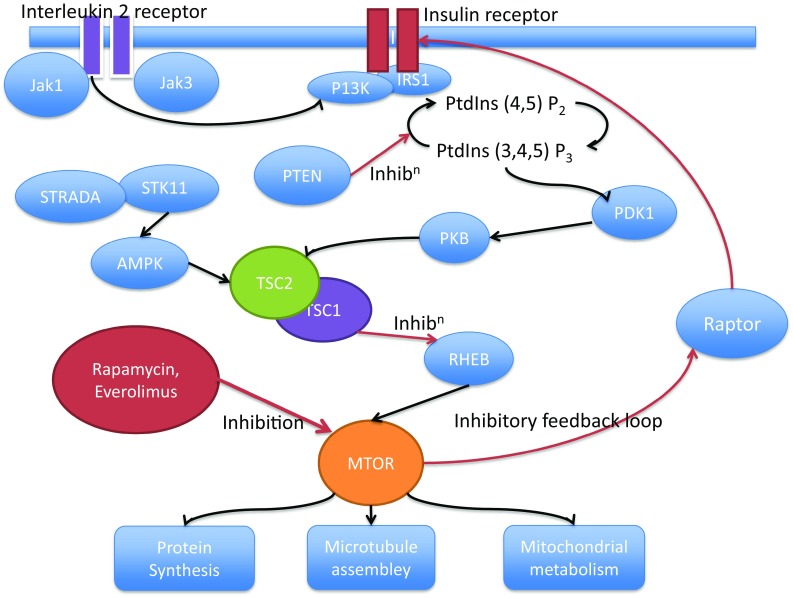
Pathway showing some of the relationships between mTOR and cellular function which may be modulated in epileptogenesis and their modulation through inflammatory pathways and by drugs. *AMPK* 5′ AMP-activated protein kinase, *IRS1* insulin receptor substrate 1, *JAK* Janus kinase, *MTOR* mechanistic target of rapamycin, *PDK1* pyruvate dehydrogenase lipoamide kinase isozyme 1, *P13K* PI3 kinase, *PKB* protein kinase B, *PtdIns* phosphatidylinositol, *PTEN* phosphatase and tensin homologue, *RHEB* ras homolog enriched in brain (GTP binding protein), *STRADA* STE20-related kinase adaptor alpha, *STK11* serine/threonine kinase 11, *TSC* tuberous sclerosis complex

Another focus is the mechanisms of epileptogenesis; the process from initiation of pathological changes to the development of epilepsy and possibly the maintenance of epilepsy. There are changes, which involve altered gene expression, inflammation, protein production and changes in connectivity, which may all be the target for drugs to suppress epileptogenesis. One of the most studied pathways links to the rapamycin (mTOR) pathway (Fig. 1). Upregulation of mTOR, a serine/threonine protein kinase, occurs as a result of the TSC1 and TSC2 mutations of tuberous sclerosis (TS) complex. Other mutations in the pathway may be associated with overgrowth in megalencephaly [80]. mTOR has a role in protein synthesis and inhibition of mTOR, cell growth and replication by everolimus, a rapamycin analogue, has been shown to reduce overgrowth of malignantly transformed tubers [81]. Animal models have shown an antiepileptic effect of mTOR inhibition [82] but this has been more difficult to demonstrate in humans. However, a recent double-blind study of 366 patients showed a dose-related seizure reduction of up to 40% with everolimus, in patients with TS [83]. However, mTOR inhibitors may also have a direct effect on Kv1.1 ion channels, independent of epileptogenesis [84], blurring their possible mechanism in seizure suppression.

Whilst immunological mechanisms are clearly implicated in the aetiology of certain epilepsies such as limbic encephalitis [85] or Rasmussen encephalitis [86], increasing attention has been given to them in commoner forms of epilepsy. There is broad evidence for their significance, especially from animal studies and involving cytokines, changes in the blood brain barrier and pathological alterations associated with altered excitability [87–94]. Pathological examination of resected human specimens of focal cortical dysplasia [95] has also shown substantial increases in mRNA expression of Toll-like receptors 2 and 4 and associated with high-mobility group box protein 1, restricted to astrocytes and microglia in pathological tissue. These interact through interleukin IL1-β. Microglia activation appears increased more in focal cortical dysplasia (FCD) type II than in FCD I, associated with the migration of activated lymphocytes and activation of the mTOR pathway, linking inflammation to epileptogenesis [96]. A recent systematic review and meta-analysis [97] has described increased CNS levels of interleukins of the IL1 family as well as of chemocytokines CCL 3-5, which are involved in monocyte and lymphocyte migration. IL6 appears to be elevated in serum but not in CNS. A recent study of patients with moderate to severe cerebral trauma found a relationship between cerebrospinal fluid IL1-β levels and an allelic variant of the IL1-β gene to the risk of developing epilepsy [98]. This provides the first evidence of a biomarker that might be used to predict epilepsy after an epileptogenic insult and possibly a means of pharmacological intervention. These may need to be complex; a recent study suggested a single intervention was inadequate and a cocktail of anti-inflammatory drugs was required to prevent epileptogenesis [99]. A small case series of intractable childhood onset epilepsy has already been treated successfully with human recombinant IL-1 receptor antagonist (Anakinra\) [100] and it is hopeful that, as there are already many drugs affecting the immune system and some affecting the blood brain barrier, that this will prove a fertile area for development.

Recently, mutations of the DEPDC5 (DEP domain containing 5, involved in g-protein signalling) gene have been demonstrated in patients with cortical dysplasia and in up to 12% of small families of patients with familial focal epilepsy phenotypes, including ADNFLE without demonstrable lesions [101–103]. This gene is involved in the same GATOR pathway as mTOR. Although the GATOR (gap activity towards RAG’s) pathway is generally associated with protein synthesis, it appears to reduce the levels of K_v_1.1 potassium channels in hippocampal pyramidal neurons increasing seizure expression, which can be reversed by inhibitors [104]. These findings link lesional and non-lesional ion channel related epilepsies to the same pathway, providing a potential opportunity for the wider use of inhibitors in treatment.

Although the scope is expanding, the relationship of these mechanisms to the majority of epilepsies, those triggered by a neurological insult (focal epilepsies) or a complex genetic trait (generalized epilepsies) remains to be established. It has long been recognized that epilepsy due to trauma is more likely in those with a family history of epilepsy [105] providing a potential to link to genetic mechanisms. But the development of epilepsy may take 20 years [105, 106]. The key will be to identify those patients at high risk and to find a low risk preventative treatment akin to aspirin in stroke and very large, long-term follow up studies, will be needed to establish efficacy. Biomarkers such as IL1-β for evolving epileptogenesis are needed to identify high risk patients and to act as drug targets.

## Antiepileptic drug trials

Despite being a common disorder, the number of high quality trials of antiepileptic drugs is small. Trials of new AED are normally in the form of an add-on therapy in refractory partial epilepsy, usually with the end point of a 50% reduction of seizures. This may be realistic in showing a biological effect but does not confer the psychosocial benefits of seizure freedom, and therefore drugs enter the market with the knowledge that they will not dramatically alter the burden of refractory epilepsy. The Federal Drug Administration in the US requires monotherapy trials against placebo and the European Medicines Agency requires head-to-head trial of active agents. Consequently, results cannot cross the Atlantic, delaying introduction and increasing cost for manufacturers. Both types of trials have their merits. The result is a non-systematic hotchpotch of evidence in relation to monotherapy in epilepsy. Whilst the pragmatic study SANAD has guided many UK clinicians to lamotrigine as first line in monotherapy for focal epilepsy [107], carbamazepine remains a drug of choice in many countries and studies [108]. A recent study has shown that zonisamide is non-inferior to carbamazepine in new onset focal epilepsy in adults [109]. A large study of 1688 new onset patients compared time to withdrawal of levetiracetam in two arms to first choice carbamazepine or valproate in monotherapy in adults [110]. Overall, the drugs performed similarly but in a post hoc analysis, levetiracetam withdrawal rate was lower in those over 60, especially in comparison to carbamazepine, with fewer adverse effects rather than greater efficacy [111].

The repertoire of AED considered effective in IGE has traditionally been more restricted that for focal epilepsy. Case reports have supported the use of lacosamide [112, 113] and it is the subject of ongoing larger scale studies. Perampanel has been found to be effective as an add-on for refractory generalized epilepsy with tonic–clonic seizures [114].

Cannabis contains approximately 80 different active cannabinoids and was used in the nineteenth century as an AED [115]. It has been known for many years to be an antagonist at NMDA receptors with antiepileptic activity [116]. Δ^9^ tetrahydrocannabinol is the main psychoactive component of cannabis, acting on THC1 and THC2 receptors but other components, especially cannabidiol (CBD) do not act on these receptors, are not psychoactive. They may have medicinal properties through a range of other actions [117]. Clinical studies in the 1970s and 80s reviewed in [117] pointed to antiepileptic effects and recent anecdotal evidence and an open labelled trial have shown benefit in epileptic encephalopathies such as Dravet syndrome [118, 119], which have had a profound social effect in the United States, with parents moving their families to states where cannabis is legal [120]. Although their mechanisms point to a potential role for cannabinoids of relevance to epilepsy [121], there are as yet, no good studies to support their widespread use. The adverse effects of natural cannabis are widely known [122] and a particular problem for adolescents. Cannabinoids should be avoided by those with epilepsy, especially the young, who are already at risk of psychiatric problems, until good quality trials support their use.

## Epilepsy and comorbid depression

Data extracted from a US population survey of 340,000 households and those with epilepsy were compared to those without [123]. Two percent had suffered with epilepsy and reported increases in a range of disorders (Table 1). A figure of approximately one third affected by depression is consistent with numerous previous studies. The relationship to epilepsy is complex. In studies of IGE, the epilepsy and its impact may be important [124] but there is often dissociation between a good seizure outcome and a poor psychosocial outcome [125]. A key factor predicting outcome relates to family environment support [126] but a biological association is supported by the observation that children and adults have an increased risk of psychiatric disturbance, even before the onset of their epilepsy [127, 128], and by a broad range of experimental studies [129]. Interactions between epilepsy and depression may include shared abnormalities in a number of neurotransmitters including 5HT_1A_ mechanisms [130, 131] and via glutamate, where low-dose ketamine, an antiepileptic NMDA antagonist, may have an impact on depression [132]. These studies illustrate a bidirectional relationship of epilepsy and depression, involving both biological and psychosocial factors.

**Table 1:** Comorbidities in a nationwide US survey [123]

	No epilepsy (%)	Epilepsy (%)
Anxiety	13.9	22.4
Depression	25.6	32.5
Bipolar disorder	6.7	14.1
ADHD	5.5	13.2
Sleep disorder/apnea	13.6	19.6
Movement disorder/tremor	4.6	9.3
Migraine	20.6	27.9
Chronic pain	17.7	25.4
Fibromyalgia	7.5	15.4
Neuropathic pain	5.6	8.7
Asthma	16.6	20.7
Diabetes	15.2	15.2
Hypertension	36.7	36.2

A common concern is that antidepressants may increase seizures. The risk of *de novo* seizures from the use antidepressants is 0.1% for newer drugs and 0.3% from older drugs, e.g. tricyclics [133]. Exceptions may be maprotiline, bupropion or clomipramine with a higher risk [134] but overall, those in the treatment arm of antidepressant trials had fewer seizures than those in the placebo arms [134]. In smaller studies of those with epilepsy at therapeutic doses of antidepressants, many will experience an improvement in their epilepsy [135]. A recent review has brought together the newer mechanistic evidence, showing that 5HT_1A_ may mediate a number of actions, which have antiepileptic effects, including increasing GABA activity and reducing inflammatory cytokines and those patients with epilepsy may have reduced PET ligand binding at 5 HT_1A_ sites [136]. In a mouse model of sudden unexplained death in epilepsy, drugs acting on 5-HT_3_ receptors (fluoxetine, blocked by ondansetron) reduced respiratory arrest in seizures, without affecting the seizures themselves [137], a further possible mode of benefit of antidepressants in epilepsy. Where possible, it may be appropriate to avoid those antidepressants with pharmacokinetic interactions with AED, such as fluvoxamine, paroxetine and fluoxetine. Hopefully, neurologists can now encourage the use of antidepressants, especially as psychiatric comorbidity is a greater determinant of quality of life than seizure frequency in those with refractory epilepsy [138].

## Antiepileptic drugs and pregnancy

In recent years, information regarding major congenital malformation (MCM) rates has been consolidated in epilepsy and pregnancy registries. AED are divided into those with reasonably quantified risk and those with insufficient data. This becomes self-reinforcing with increased reluctance to prescribe drugs of uncertain risk to those who may conceive. The most recent data from the UK epilepsy and pregnancy register, shows a very clear dose-related effect with valproate risk 5% with <600 mg daily increasing to 11% at over 1000 mg. Carbamazepine at 2% risk when given at <500 mg daily, 3% at 500–1000 mg and 5% at >1000 mg. Lamotrigine had a less steep curve with 2% at <200 mg, increasing to 3.5% over 400 mg daily [139]. These data are similar to those published from European and US registries [140]. Oxcarbazepine, not widely used in the UK, appears to have similar low risk to lamotrigine at 2.2% [141]. The risk for levetiracetam appears similarly low at 0.7% in monotherapy, increasing in polytherapy [142]. Added to the risk of MCM are concerns over more subtle neurodevelopmental disturbances, including lower IQ, autism and ADHD, which may conceivably arise from exposure to valproate at any stage of pregnancy [143–146]. Although not widely used in pregnant women, topiramate and zonisamide may be associated with significantly lower birthweight [147]. Recent data have also shown the importance of considering genetic factors in teratogenicity. A family history of abnormalities increases the risk. The risk to a second child, where a first was affected by an AED may be as high as 17–36% [148, 149]. Clinicians must also consider the risk to the mother of epilepsy in pregnancy and data suggest a tenfold increase in mortality compared to non-epilepsy controls, largely due to SUDEP [150].

## Epilepsy surgery

Given the low chance of response to medical therapy after the failure of two AED [49], this is the widely accepted yardstick for defining refractoriness and the appropriateness for consideration of resective epilepsy surgery. The proportion of patients for whom surgery may be successful is not clear, but is estimated as a maximum of around 2% of the total cohort. With an incidence of 0.5%, in the USA and a prevalence of 750,000, this translates to up to 3500 incident cases and 15,000 prevalent cases, in which surgery might be considered. The rate of epilepsy surgery has remained static at around 1500 cases per year [151, 152] for over 20 years. The pattern of cases operated may be changing with a reduction in mesial temporal sclerosis [153], perhaps due to improved outcomes of childhood febrile seizures. At the same time, the outcomes of extratemporal epilepsies are improving with new diagnostic techniques. The mortality of surgery is around 0.1–0.5% [151], similar to the annual rate of SUDEP in refractory epilepsy [154], i.e. the mortality of ongoing refractory epilepsy exceeds the post-operative risk after one year. Complication rates have reduced [155] and are around 3% for major and 7% for minor complications; one of the commonest complications is a visual field defect after temporal lobectomy [151, 156]. The treatment is cost-effective in the long term, with sustained remission and close to half of adult patients and 86% of children may be able to stop their AEDs. Two recent studies have found risk factors for seizure recurrence after post-operative drug withdrawal included pre-operative seizure frequency and post-operative EEG abnormalities [157, 158]. They also found about one third of those relapsing will not come back under control with re-introduction of medication, especially those with early recurrence, perhaps reflecting a less complete surgical remission.

Health-related quality of life often returns to normal in those who become seizure free [159]. Negative prognostic factors include high seizure frequency and long duration at baseline [160, 161]. Those with lesions such as cavernomas or benign tumours may achieve 77% seizure freedom at two years, even if surgery is undertaken after a long seizure history [162].

Advances in epilepsy surgery include alternative methods to resective surgery; improvements in techniques of case selection for surgery and neurostimulation techniques.

Radiosurgery for arteriovenous malformations may give excellent outcomes for associated epilepsy and positive prognostic factors have been reported to be presentation with haemorrhage rather than epilepsy and the absence of post-treatment haemorrhage [163–165]. A recent meta-analysis of stereotactic radiosurgery for mesial temporal sclerosis [166] showed that the total number of patients reported remains low (<200) but that half became seizure free at a median of 14 months after treatment with a complication rate of around 8% (excluding headache which was more common) and rates of visual field defects similar to open surgery. MRI-guided laser thermocoagulation has been undertaken in a few patient with initially promising results. Procedural morbidity is low and patients may be admitted for just one day. It has been suggested as appropriate particularly for older patients. [167–170]. The electrodes inserted for stereotactic EEG recording may also be used to deliver a thermocoagulation induced lesion to the surrounding brain, with a diameter of 4.5–7 mm. This has been undertaken in patients with hypothalamic hamartoma, for whom surgery is difficult and with a high success in remission of the gelastic seizures associated with these lesions [171]. Early indications are that this may be an approach which can be undertaken in cases of focal cortical dysplasia.

The identification of patients who will benefit from epilepsy surgery relies on the demonstration of a single brain region responsible for the epilepsy, which can be safely resectable. Identification of a responsible lesion has been demonstrated in numerous studies to predict a better outcome [172]. Even in those where imaging is normal, resection on the basis of an intracranial EEG abnormality is more likely to result in seizure freedom if the resected tissue is pathologically abnormal [173]. Increasing pre-operative identification of pathology through improved MRI, through higher field strengths up to 7 T in vivo and enhancing 3 T with automated measures of hippocampal volumes potentially gives a greater chance of identifying candidates who may benefit from surgery [174–176]. In those whom structural imaging remains negative, then FDG-PET can aid in the decision making, either in favour of surgery, e.g. in those thought to have non-dominant TLE or against surgery in more complex cases [177, 178]. Magnetoencephalography is not widely used [179], but a recent study demonstrated that if all MEG abnormal areas were resected, prognosis was improved and MEG can be used to target SEEG more successfully [180]. Tight clustering of MEG abnormalities predicted a better outcome than more dispersed abnormalities. High density EEG source imaging using increased electrode number may also be valuable in predicting the outcome of surgery [181]. Intravascular stent EEG, shown to be safe in sheep may be a non-invasive method of intracranial EEG recording in the future [182].

Where resective surgery is not possible, palliative stimulation techniques may be considered. The most established and widely used is vagus nerve stimulation which is safe, with a low risk of complications, such as infection, haematoma and vocal cord palsy [183]. An analysis from the VNS registry combined with pooled study data totaling 8423 patients [184] found that responder rate, defined by a 50% seizure reduction, was 47% at 0–14 months and 63% at 24–48 months with seizure free rates rising from 5–10% over the same period. Quality of life measures also improved with VNS [185], which may relate to seizure reduction, reduced AED load in association with successful antiepileptic treatment or putative effects of VNS on mood [186]. Responsive stimulation involves a closed circuit of intracranial electrodes with electrical stimuli delivered to the brain according to a seizure detection paradigm. The circuit is often installed following electrode placement in an unsuccessful attempt to identify a surgical target. In 191 patients there was a 37.9% responder rate compared to 17.3% in the sham group. [187]. Electrodes placed in the thalamus have been associated with a 69% median reduction in seizure frequency and a 35% rate of serious adverse events, including infection in 10% and lead misplacement in 8% [188]. Other targets under investigation include the nucleus accumbens [189] and the cerebellum [190]. Optogenetic methods [191], successful in animals, have not yet been applied in humans.

## Summary

A new classification of epilepsies will support the integration of novel aetiological and genetic factors with the existing electroclinical classification and help identify when a single seizure might be considered epilepsy on the basis of an abnormal EEG or imaging. Midazolam IM has emerged as the benzodiazepine of choice in out-of-hospital treatment of status epilepticus and a valid alternative in hospital, but good clinical studies are lacking beyond this early stage. Limbic encephalitis is increasingly diagnosed and primary treatment is immunotherapy rather than AED. The significance of antibodies more generally in epilepsy remains unclear. Most epilepsy treatment remains without a clear evidence base but ethosuximide and valproate have been demonstrated to be the most efficacious AED in absence epilepsy. Perampanel and lacosamide are new drugs which are emerging as treatments for tonic–clonic seizures in generalized epilepsy. A small number of specific genetic epilepsies have allowed personalized treatment in specific cases but this has not yet had broader application. Epileptogenesis is a fertile area of research and everolimus, an inhibitor of the mTor pathway, has demonstrated efficacy in epilepsy associated with TS, showing the clinical potential of this avenue of research for the first time. Epilepsy and pregnancy registers are consolidating data pointing to the use of lamotrigine, levetiracetam, carbamazepine and/or oxcarbazepine as those AED with the lowest risk of major congenital malformations. New evidence has associated topiramate and zonisamide with low birth weight. Clinicians can treat comorbid depression with most modern antidepressants, reassured that there is little evidence of an adverse effect on their patient’s epilepsy. Surgical treatment of epilepsy remains under-utilised and the selection of patients for surgical treatment of epilepsy is becoming more refined with the use of functional imaging to support structural imaging. Alternative ablative treatments are being explored but are not yet widespread. Stimulation techniques other than VNS are areas of research, which remain to find their place.

Overall, recent epilepsy research has started to change our thinking and approach to patients, as we slowly move towards a more rational basis by which to treat this common condition.
